# Effective Repeated Production of γ-glutamylcysteine, Essential For Intracellular Glutathione Production, Using Cellulose-immobilized Phytochelatin Synthase-like Enzyme NsPCS

**DOI:** 10.1007/s12010-024-05137-5

**Published:** 2025-01-21

**Authors:** Takuya Nakai, Kazumasa Hirata, Kazuya Nagano, Masayoshi Arai, Hiroshi Uyama, Yoshihiko Hirata, Misa Muraoka

**Affiliations:** 1 Graduate School of Pharmaceutical Sciences, Osaka University, Suita Yamadaoka 1-6, Suita, Osaka 565-0871 Japan; 2 School of Pharmaceutical Sciences, Wakayama Medical University, Shichibancho 25-1, Wakayama, 640-8156 Japan; 3 Graduate School of Engineering, Osaka University, Suita Yamadaoka 2-1, Suita, Osaka 565-0871 Japan; 4 Biochemical Laboratory, Saraya Co., Ltd., 24-12 Tamate-Cho, Kashiwara, Osaka 582-0028 Japan

**Keywords:** γ-glutamylcysteine, Glutathione, Phytochelatin synthase like enzyme, Enzyme immobilization, Cellulose carrier, Stable and low cost production

## Abstract

γ-Glutamylcysteine (γ-EC) can increase intracellular glutathione (GSH) levels, which may prevent and alleviate age-related disorders and chronic diseases caused by oxidative damage. However, the commercial availability of γ-EC remains limited owing to its complex chemical synthesis from glutamate and cysteine. In this study, we have developed the method of the effective conversion of GSH to γ-EC to achieve the optimal reaction conditions for repeated batch production and potential application in industrial γ-EC production using the phytochelatin synthase-like enzyme NsPCS. For repeated batch conversion reactions, the optimal temperature was determined at 25 °C, where γ-EC showed good stability compared with that at 37 °C, leading to higher overall productivity. Cellulose sponges and microcrystalline cellulose (MCC) showed superior mechanical strength as immobilization carriers and greater stability and productivity than other materials. The total amounts of γ-EC obtained by NsPCS immobilized on the cellulose sponge and MCC were 305 mg and 291 mg, respectively, in a 5 mL reaction over five repeated batch reactions. These simple production processes are easily reproduced, and their high volumetric efficiency is promising for the industrial production of stable and low-cost γ-EC.

## Introduction

**Fig. 1:**
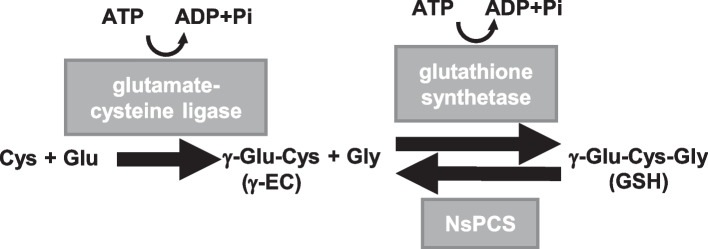
Reaction pathways of glutamate–cysteine ligase, glutathione synthetase, and phytochelatin synthase-like enzyme derived from *Nostoc* sp. Pasteur Culture Collection 7120 (NsPCS)

Glutathione (GSH) can prevent intracellular oxidative damage caused by the generation of reactive oxygen species, free radicals, and peroxides [1, 2]. Therefore, decreasing intracellular levels of GSH are associated with many diseases, such as age-related disorders and chronic diseases caused by oxidative stress [3]. Intracellular GSH is synthesized through two reactions as shown in Fig. 1. First, γ-glutamylcysteine ligase (GCL) catalyzes the synthesis of γ-glutamylcysteine (γ-EC) from glutamate (Glu) and cysteine (Cys). Second, glutathione synthetase (GS) catalyzes GSH synthesis from γ-EC and glycine (Gly). In these sequential ATP requiring reactions, the first reaction is rate-limiting and the activity of the GCL is elaborately regulated by intracellular GSH level to maintain GSH homeostasis. GS generally shows higher activity than GCL and no inhibitory regulation. In age-related disorders and chronic diseases including Alzheimer’s disease (AD), decreasing intracellular GSH levels is mainly attributed to declining GCL activity [4, 5]. In humans, passive transport of GSH from the plasma into the cell is not thermodynamically favorable, because intracellular GSH levels are much higher than the plasma [6]. GSH has been demonstrated to be not actively transported into the cell except only a few cell types, notably kidney and intestinal epithelial cells [7, 8]. In particular, Witschi et al*.* reported exogenously supplied GSH was not effective in increasing GSH levels in cells with dysfunctional GCL [9]. Administrated of *N*-acetylcysteine (NAC), a chemically synthesized prodrug of Cys, is used to increase intracellular GSH levels, because NAC is easily transported into cells and successively converted to Cys and GSH through γ-EC [10]. However, NAC is likely ineffective in increasing GSH levels in cells with dysfunctional GCL, in which γ-EC synthesis from Cys converted from NAC would be suppressed [5].

Since γ-EC is an intermediate precursor of GSH (Fig. 1), intracellular supplementation with γ-EC is expected to increase GSH levels [11–13], thereby maintaining and enhancing the antioxidative functions of GSH. Indeed, several previous studies reported an increase in intracellular GSH levels following γ-EC administration. Liu et al*.* [14] reported that the oral administration of γ-EC increased neuronal GSH levels in a mouse model of AD, consequently reducing neurodegeneration and ameliorating cognitive decline. γ-EC also exerted neuroprotective effects in AD [15]. Therefore, γ-EC is expected to contribute to prevention and improvement of age-related disorders and chronic diseases, in particular for neurodegenerative diseases by increasing intracellular GSH level.

However, γ-EC does not accumulate intracellularly in most species, because GCL catalyzing γ-EC synthesis is a rate-limiting enzyme and synthesized γ-EC is immediately converted to GSH by GS (Fig. 1). Therefore, it is difficult to biosynthesize and accumulate γ-EC using a fermentation process, such as is generally used in the industrial production of amino acids and peptides. The current method of γ-EC production is mainly chemical synthesis from Glu and Cys. However, this chemical process includes complex reaction processes and low yield, resulting in high cost and low purity of γ-EC, limiting the production of γ-EC for physical function investigations and medical/pharmaceutical applications. For example, commercially available γ-EC reagents are < 100 mg and 3,700 US dollars (USD) per gram or more expensive. To establish a γ-EC production process with high productivity and low-cost, we have developed a novel enzymatic production process in which γ-EC is directly synthesized from GSH using the phytochelatin synthase like enzyme NsPCS derived from the freshwater cyanobacterium *Nostoc* sp. (Fig. 1) [16]. This enzyme efficiently converted GSH to γ-EC and glycine with high yield even at high GSH concentrations, such as 100 mM. We also effectively applied NsPCS covalently immobilized on cellulose materials in the small-scale high-yield conversion of GSH to γ-EC [17]. In this study, we elucidated the effects of temperature and immobilization carriers on the conversion of GSH to γ-EC and its yield to achieve the optimal reaction conditions for repeated batch production and potential application in industrial γ-EC production.

## Materials and Methods

### NsPCS Expression and Purification

The crude NsPCS preparation was outsourced to Protein Express Co. Ltd. (Chiba, Japan). *Escherichia coli* C43 (DE3) (Lucigen, Middleton, WI, USA) was used as the host and pET-25b-alr0975 (prepared in our laboratory) was used as the plasmid vector. Plasmid vectors were prepared as previously described [18]. The recombinants were pre-cultured in Luria–Bertani medium (1% tryptone, 0.5% yeast extract, and 0.5% NaCl) for 18 h at 37 °C. The main culture was conducted in Terrific Broth medium [47.6 g/L (Invitrogen, Tokyo, Japan), and 0.4% glycerol] and 1% of the pre-culture inoculum. The induction of NsPCS expression was performed via isopropyl β-D-thiogalactopyranoside stimulation (final concentration of 0.5 mM) and incubation at 20 °C for 20 h. After the main culture was completed, the culture medium was collected, 200 mL of Buffer A, 50 mM Tris–HCl buffer (pH 8.0) including 100 mM NaCl, 1 mM DDT, and 1 mM EDTA, was added to the bacterial pellet, and the pellet was lysed by ultrasonication. The disrupted cell liquid was centrifuged at 18 000 g for 30 min and the supernatants were collected as the soluble fraction.

The soluble fraction was sterilized using a 0.22 μm filter and treated with HiTrap Q HP (5 mL; Cytiva, Tokyo, Japan) pre-equilibrated with Buffer A. HiTrap Q HP was washed with 20 mL of Buffer A to elute the NsPCSs, followed by 20 mL of Buffer B, 50 mM Tris–HCl buffer (pH 8.0) including 1,000 mM NaCl, 1 mM DDT, and 1 mM EDTA, to elute contaminant proteins. Because the removal of contaminant proteins was insufficient, the fraction eluted by Buffer A was dialyzed into Buffer C, 50 mM Tris–HCl buffer (pH 8.0) including 10 mM NaCl, 1 mM DDT, and 1 mM EDTA, to reduce the salt concentration and treated with HiTrap Q HP (5 mL) pre-equilibrated with Buffer C. After elution of NsPCS with 20 mL of Buffer C, contaminant proteins were eluted with 20 mL of Buffer B. Because the removal of contaminant proteins was insufficient, even when the salt concentration was lowered, the flow-through fraction was repeatedly applied up to four times. The resulting flow-through fractions were concentrated in Amicon 10 K (Merck, Darmstadt, Germany) and dialyzed in Buffer D, 50 mM Tris–HCl buffer (pH 8.0) including 100 mM NaCl, 5 mM tris (2-carboxyethyl) phosphine hydrochloride (TCEP), and 25% glycerol. The insoluble components precipitated and the supernatant was collected by centrifugation and used as purified NsPCS (22 mL) in immobilization and γ-EC production.

### NsPCS Immobilization

The immobilization carriers were prepared as follows: Cellulose sponges (Shinwa rules, Nigata, Japan) were cut into 0.5 cm squares and used as sponges. Microcrystalline cellulose (MCC) was used for column chromatography (Merck, Darmstadt, Germany; particle size: > 100 μm, density: 0.07–0.40 g/cc). Quantitative Ashless No. 2 cellulose filter paper (Advantec, Tokyo, Japan) was cut into 5-mm diameter circles. In addition to the filter paper, the same diameter circle sheets of cellulose cooking paper (Lion, Tokyo, Japan) and unwoven sheet cut out from surgical mask 7–3622-01 (As One Corporation, Osaka, Japan) were used for immobilization as sheet-type carrier.

The immobilization of NsPCS followed Muraoka et al*.* [17]. About 100 mg (dry weight) of immobilization carriers were collected and weighed in a 15 mL tube, *N,N*-dimethyl-4-aminopyridine (DMAP) (FUJIFILM Wako Pure Chemical Corporation, Osaka, Japan) and *N,N'*-disuccinimidyl carbonate (DSC) (Tokyo Chemical Industry, Tokyo, Japan) were weighed to 1.51- and 3.16-times the weight of the immobilization carrier, respectively. Superdehydrated acetonitrile (10 mL, FUJIFILM) was added and stirred to completely dissolve DMAP and DSC, and then shaken for 24 h at room temperature using rocker shaker MR-1 (Biosan, Riga, Latvia). After 24 h of reaction, the immobilization carriers were washed three times with 2 mL of superdehydrated acetonitrile, and the superdehydrated acetonitrile was completely volatilized in a centrifugal vacuum concentrator at 30 °C. The immobilized carriers were washed once with 2 mL of 20 mM potassium phosphate buffer (pH 8.0).

NsPCS were dissolved in Milli-Q water and adjusted to a concentration of 220 mg/L. A portion of the sample was collected as a pre-immobilization sample, and the immobilization carriers were immersed in 4 mL of the NsPCS solution for 30 min under ice-cold conditions. After 30 min, a portion of the NsPCS solution and immobilization carriers were collected. The immobilization enzymes were washed three times with 2 mL of 20 mM potassium phosphate buffer (pH 8.0).

The protein concentrations were determined as follows: Bovine serum albumin (BSA) was dissolved in Milli-Q water at 25, 50, 100, and 150 mg/L. Protein Assay CBB Solution (5 ×) (Nacalai Tesque, Kyoto, Japan) was diluted fivefold with ion-exchanged water and dispensed in 1.5 mL portions into 2 mL tubes. The solution was diluted two-fold with ion-exchange water; ion-exchange water, BSA standard, and 75 μL of the two-fold diluted NsPCS sample were added and stirred by vortexing; and the sample for analysis was allowed to stand at room temperature for 10 min. The samples were centrifuged at 1000 rpm for 3 min using a KUBOTA 3520 (KUBOTA, Tokyo, Japan), and absorbance was measured at 595 nm using a GeneQuant 1300 spectrophotometer (Biochrome, Cambridge, UK). The NsPCS concentrations were calculated based on a calibration curve prepared using the BSA standard.

### Enzymatic Reaction Conditions

The substrate solution used for the NsPCS reaction was prepared as follows: 28.7 mg of tris (2-carboxyethyl) phosphine hydrochloride (TCEP) (Tokyo Chemical Industry) and 3.0733 g of GSH (Nacalai Tesque) were weighed in a 100 mL volumetric flask, 20 mL of 1000 mM potassium phosphate buffer (pH 8.0) was added, and the solution was gently shaken. After the addition of ion-exchanged water to the marked line, the solution was shaken to complete homogeneity. The solutions were dispensed in 5 mL portions into 19 15-mL-tubes and stored frozen at –30 °C. The frozen stock was thawed immediately before use and the reaction was initiated by adding an immobilized enzyme. Enzymatic reactions were performed in an incubator (SANYO MIR-153) (SANYO Electric, Osaka, Japan) at 37 °C or 25 °C and rotated at 100 rpm on a TAITEC NR-2 rotary shaker (Saitama, Japan).

### High-performance Liquid Chromatography Analysis

A LaChrom ELITE system (Hitachi High-Tech Corporation, Tokyo, Japan) was used for the high-performance liquid chromatography (HPLC) analysis, with a L2100 pump, L2200 autosampler, L2300 column oven, and L2455 detector. The analytical method followed that of Muraoka et al*.* [17]. The eluent was prepared as follows: Eluent A was prepared by adding 15 mL of 500 mM 1-octanesulfonic acid sodium salt solution and 0.2 mL of trifluoroacetic acid to an appropriate volume of distilled water for HPLC (Nacalai Tesque), made up to a total of 1000 mL. Eluent B was prepared by adding 15 mL of 500 mM 1-octanesulfonic acid sodium salt solution (Nacalai Tesque) and 0.2 mL of trifluoroacetic acid (Nacalai Tesque) to 300 mL of acetonitrile for HPLC (Nacalai Tesque), with a total volume of 1000 mL made up with distilled water for HPLC (Nacalai Tesque). The HPLC analysis was performed at a flow rate of 1.4 mL/min. The volume ratio of the eluents A and B, A/B, was changed linearly from 100%/0% to 0%/100% during 0 to 16 min, kept 0%/100% during 16 min to 23 min, and then changed fast linearly from 0%/100% to 100%/0% during the next 1 min. After that, at 24 min, 100%/0% was kept until 27 min. We used a Inertsil ODS-3 column (5 µm, 4.6 × 250 mm; GL Science, Tokyo, Japan) with a temperature setting of 40 ± 1 °C. Each sample was diluted to the appropriate concentration with 1 mM HCl and then injected at 50 μL. The detection wavelength was set at 215 nm. GSH and γ-EC standards (Sigma-Aldrich, St. Louis, MO, USA) were used for determining their respective concentrations.

### Statistical Analysis

Data are presented as the mean ± standard deviation (SD). In the evaluation of immobilization carriers and of the effects of temperature, Tukey’s test was used to identify significant differences between groups. The threshold for significance was set at *p* < 0.05.

## Results and Discussion

Decreasing intracellular GSH levels are associated with age-related disorders and chronic diseases caused by oxidative stress [3]. Supplement of GSH is ineffective to increase its intracellular level, because passive and active transportations of GSH into cells are not expected. Administration of NAC is also ineffective, because this prodrug of Cys is not expected to convert to GSH in the cells of the diseases where GCL activity declines. On the other hand, there have been several reports administration of γ-EC was effective to increase intracellular GSH levels [5]. Therefore, γ-EC represents a promising agent in preventing and improving age-related disorders and chronic diseases caused by oxidative stress. However, γ-EC is difficult and expensive to produce at commercial levels. There have been many challenges to develop alternative production method of γ-EC with high productivity and low cost.

We found that high concentration of GSH is hydrolyzed to γ-EC and Gly by a novel enzyme, NsPCS [16]. Immobilized enzyme is thought to be more cost effective than free enzyme because of its reusability. NsPCS is preferable for the application of this technique, because this enzyme can efficiently convert GSH to γ-EC without any additives [18, 19]. Therefore, we investigated the production process of γ-EC by using immobilized NsPCS.

In our previous study, the highest conversion rate of GSH and yield of γ-EC were obtained at 37 °C in free NsPCS. Both GSH and γ-EC are instable during enzyme reaction, because their strong reducing powers lead to easy oxidation under reaction. Deterioration of enzyme is usually dependent on reaction temperature. Therefore, 37 °C is thought be not sufficient for the stability of GSH, γ-EC, and even NsPCS, indicating optimal temperature for the reaction using immobilized NsPCS must be investigated for stable and high-yield production of γ-EC.

**Fig. 2:**
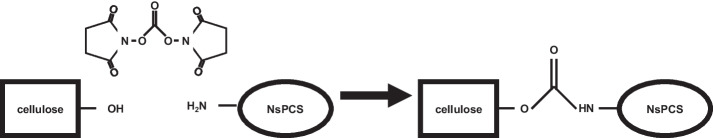
The mechanism of NsPCS immobilization of cellulose carrier. Carrier cellulose is activated with DSC in the presence of DMAP

To apply immobilization to NsPCS, we examined, the availability of several representative immobilization techniques. Among them, a covalent binding technique between cellulose carrier and NsPCS using DSC and DMAP showed the highest stability and γ-EC yield [17]. This technique has been developed and already applied to horseradish peroxidase [20]. Figure 2 showed activation of cellulose and immobilization with NsPCS, in which carrier cellulose was activated with DSC in the presence of DMAP. Cellulose filter paper was employed first as an immobilization carrier because of it commonly available and easy to apply for batch production. However, our current studies revealed that mechanical strength of filter paper was insufficient for repeated use, because severe fiber crumbling occurred during repeated reactions (data not shown). For repeated use of immobilized NsPCS, therefore, alternative adequate carrier with high stability must be required for repeated use.

In this study, we investigated optimal temperature and immobilization carrier for conversion of GSH and γ-EC in repeated batch production process.

The conversion of GSH and yield of γ-EC were calculated as follows, given that the degradation of GSH and γ-EC during reactions was reduced. *GSH*_*0*_ and γ-*EC*_*0*_ were the initial concentrations, while *GSH*_*t*_ and γ-*EC*_*t*_ were the concentrations after incubation for *t* h.

$$Conversion\;of\;GSH\left({\%}\right)=\frac{{GSH}_{0}-{GSH}_{t}}{{GSH}_{0}}\times 100$$

$$Yield\;of\;\gamma EC \left({\%}\right)=\frac{{\gamma EC}_{t}-{\gamma EC}_{0}}{{GSH}_{0}}\times 0.66\times 100$$

A purchase γ-EC was used as a standard for HPLC analysis to determine γ-EC concentrations in the reaction solution. The purity of this chemically synthesized reagent was shown as > 80% but the accurate purity was unknown. In parallel with this investigation, we synthesized γ-EC from GSH by NsPCS in 500 mL scale reaction and purified by anion-exchange resin column. The purity of γ-EC obtained was 96% on weight-based calculation with 4% impurity from phosphate used as buffer for reaction. The purity of the standard γ-EC was estimated to 66% from the comparison on HPLC analysis between the standard and the purified γ-EC. Consequently, the yield was calculated using this purity, 66%, from the concentrations of γ-EC determined by HPLC analysis using the standard γ-EC with assumed purity, 100%.

Cellulose is a common immobilization carrier for enzymes and microorganisms in biochemical synthesis [21, 22] Various materials, such as sheet-type and porous-type materials, are available at low cost and easily reshaped. Therefore, the immobilization of NsPCS on cellulose carriers represents a promising alternative for stable and cost-effective γ-EC production from GSH. In the current study, we applied a porous cellulose sponge as an immobilization carrier, because its cellulose fibers were thought to be molded more stably and had greater mechanical strength than the filter paper.

**Fig. 3:**
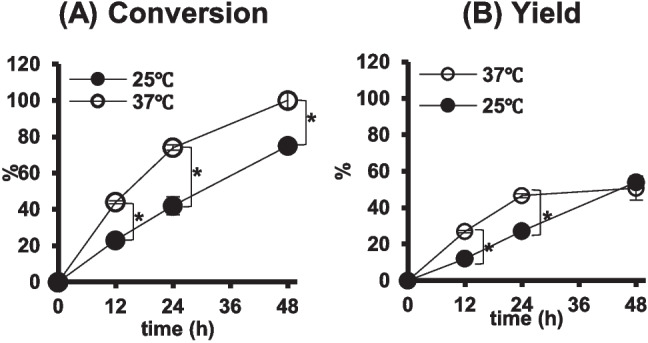
The effect of reaction temperature on conversion of GSH and yield of γ-EC of NsPCS immobilized on cellulose sponge. Glutathione at 100 mM was converted by immobilized NsPCS on cellulose sponge at 25 °C (●) and 37 °C (〇) in 5 mL reaction volume. The conversion of GSH and the yield of γ-EC were examined at 12, 24, and 48 h after the reaction (*N* = 3). Mean values are shown as line graphs and standard deviations are shown as error bars. * *P* < 0.05, Tukey’s test

At first, we compared the effect of 25 °C on the conversion of GSH and the yield of γ-EC to 37 °C by using NsPCS immobilized on the cellulose sponge. At 25 °C, the conversion of 100 mM GSH was incomplete after 48 h and significantly poorer than that at 37 °C, in which almost complete conversion was obtained (Fig. 3A). Although the yield of γ-EC at 25 °C during < 24 h of reaction were also lower than those at 37 °C, the yield at 25 °C increased almost linearly and in parallel with increase of conversion of GSH and reached approximately the same value, 54%, as that observed at 37 °C after 48 h (Fig. 3B). These results indicated that GSH and γ-EC were more stable at 25 °C than at 37 °C, suggesting that higher productivity of γ-EC may be obtained at 25 °C than at 37 °C, despite the reduced reaction rate.

**Fig. 4:**
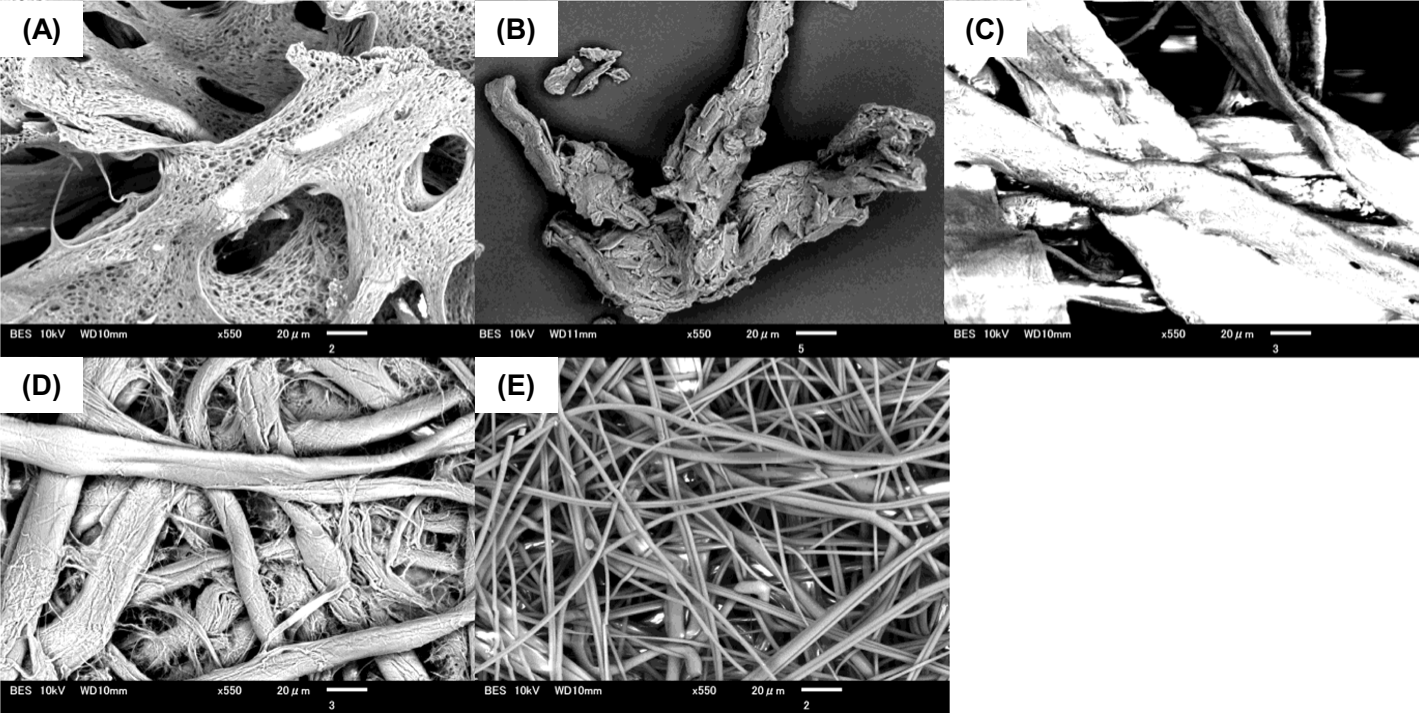
Scanning electron microscope (SEM) images of the immobilization carriers. (**A**) cellulose sponge, (**B**) MCC, (**C**) cellulose cooking paper, (**D**) cellulose filter paper, and (**E**) unwoven sheet

Second, we compared the conversion of GSH and yield of γ-EC by NsPCS immobilized on cellulose sponge with those in other carrier materials, MCC, filter paper, cooking paper, and unwoven sheet. The scanning electron microscope (SEM) images of the immobilization carriers were shown in Fig. 4.

**Fig. 5:**
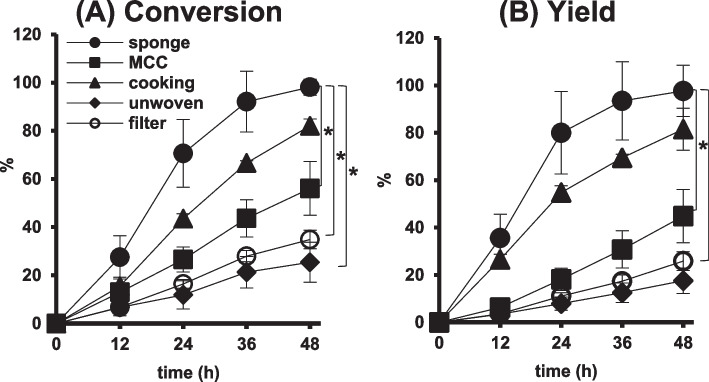
Effect of immobilization carriers for NsPCS on conversion of GSH and yield of γ-EC. Glutatione at 100 mM was converted at 25 °C in 5 mL reaction volume by immobilized NsPCS on five different carriers: sponge (●), MCC (■), cooking paper (▲), unwoven sheet (◆), and filter paper (〇). The conversion of GSH (A) and yield of γ-EC (B) were examined at 12, 24, 36, and 48 h after the reaction (*N* = 3). Mean values are shown as line graphs and standard deviations are shown as error bars. * *P* < 0.05, Tukey’s test

As shown in Fig. 5A, an almost complete and the highest conversion of GSH among the materials was obtained after 48 h in NsPCS immobilized on the cellulose sponge. In comparison with filter paper used in our previous study, higher conversion rates were observed in the carriers of cellulose cooking paper and MCC. The conversion rates were lowest in unwoven sheets. Time-course profiles of the yield of γ-EC in individual carriers were similar to those of the conversion of GSH (Fig. 5B). The sponge and MCC showed strong mechanical strengths and maintained their original shapes. On the other hand, serious fiber crumbling was observed in the cooking paper, similar to that in the filter paper. These results suggested that cellulose sponge is the most adequate immobilization carrier to achieve high productivity in a one-time batch reaction. Indeed, in SEM image of cellulose sponge, more porous microstructure was observed in comparison with those of other materials. This observation indicates that the sponge has a greater specific surface area, thereby enabling it to accelerate the conversion reaction of GSH to γ-EC by NsPCS. All of the SEM images of the immobilization carriers except MCC, exhibited the presence of a fibrous structure, which was not observed in MCC.

**Fig. 6:**
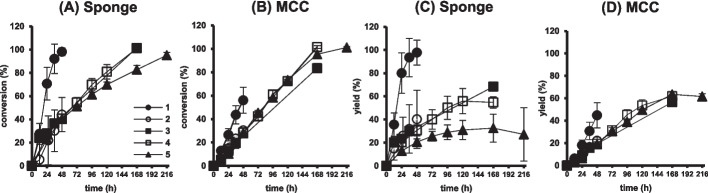
Repeated production of γ-EC by NsPCSs immobilized on cellulose sponge and MCC. Glutathione at 100 mM was converted by NsPCS immobilized on cellulose sponge (**A**, **C**) and MCC (**B**, **D**) at 25 °C in 5 mL reaction volume over five repeated reactions. The conversion of GSH (**A**, **C**) and yield of γ-EC (B, D) were examined at the indicated reaction times in the first (●), second (〇), third (■), fourth (□), and fifth (▲) reactions (*N* = 3). Mean values are shown as line graphs and standard deviations are shown as error bars

Third, repeated batch conversion reactions were performed using the NsPCSs immobilized on cellulose sponge and MCC. In the first reaction with the cellulose sponge, an approximately complete conversion of GSH (Fig. 6A) and equivalent γ-EC yield, 98%, were obtained after 48 h (Fig. 6C). However, in the second reaction, the conversion and yield were significantly reduced, and the reaction was incomplete after 48 h. Therefore, the reaction periods were extended to 168 h in the third and fourth reactions and 216 h in the fifth reaction. In the third and fourth reactions, the reaction rates did not differ, and the conversion of GSH was almost complete (Fig. 6A). However, during the fifth reaction, the reaction and conversion rates were lower than those before. For the NsPCSs immobilized on the cellulose sponge, the γ-EC yield was reduced with repeated reactions: In the fifth reaction, the yield after 48 h was reduced to 26% of the first reaction (Fig. 6C). For the NsPCSs immobilized on MCC, the reaction and conversion rates were approximately half those of the cellulose sponge in the first reaction (Fig. 6B). From the second to fifth repeated reactions, the initial reaction rates were lower than those of the sponge; however, the rates remained stable during each reaction, and the conversions were comparable to those of the sponge. The γ-EC yield in the third to fifth reactions were comparable to and, eventually, significantly higher than those of the sponge (Fig. 6D). For the cellulose sponge, the reaction rate was significantly higher than that for MCC; however, the γ-EC yields were unstable and decreased with repeated reactions. In contrast, the MCC yield remained stable until the fifth reaction.

**Fig. 7:**
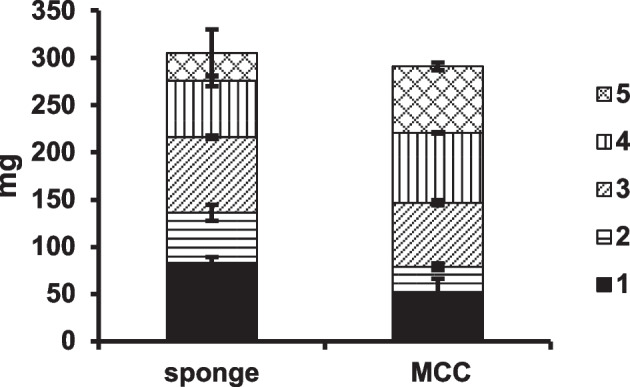
Productivity of γ-EC in repeated reactions by NsPCS immobilized on cellulose sponge and MCC. Glutathione at 100 mM was converted by NsPCS immobilized on cellulose sponge or MCC at 25 °C in 5 mL reaction volume through five repeated reactions. The total amount of γ-EC was obtained by summing the yields of the first (■), second (▤), third (▧), fourth (▥), and fifth (▩) reactions. The standard deviations are shown as error bars (*N* = 3)

Figure 7 shows the productivity of γ-EC in the repeated five-batch reactions using NsPCSs immobilized on the cellulose sponge and MCC. The reaction time was 48 h for first and second reactions, 168 h and 216 h for the third and fourth reactions and the fifth reaction, respectively. The reaction volume (5 mL) contained 0.1 g of sponge or MCC. The total amount of γ-EC obtained by 5 times repeated reactions in the cellulose sponge was 305 mg, comparable with the 291 mg of MCC, which was more than three times higher than those of the filter paper used in our previous study under the same conditions (data not shown). The current market price for 305 mg γ-EC is approximately 1,100 USD.

We used a 5-mL reaction solution in our experiments. In this scale, we consumed 10 USD for immobilization of NsPCS on sponge and 40 cents for making 5-mL substrate solution. Thus, the reagent grade 5-repeated batch reaction of NsPCS immobilized on sponge required 12 USD.

Fortunately, the industrial production of γ-EC from GSH using immobilized NsPCS in large-scale systems does not require more complex reaction conditions. For example, from a 1,000-mL reaction volume in an open container, such as a beaker or flask, with constant low-rate stirring, 100 mM γ-EC (equivalent to 25 g per 1,000 mL) is ideally obtained from the same concentration of GSH with high volumetric efficiency. Additionally, an increase in the number of containers can increase productivity without scaling up the reaction device. In this study, the reaction volume was increased to 5 mL from the 100 μL used in our previous study at 100 mM GSH to confirm the feasibility of large-scale production using simple 1,000-mL rection systems, for example.

In conclusion, the reaction temperature, 25 °C, was more sufficient than 37 °C for γ-EC production by immobilized NsPCS. Cellulose sponge is an adequate immobilization carrier for γ-EC production; however, the reaction rate and yield of γ-EC decreased considerably during repeated reactions. The substrate GSH was newly supplied and the synthesized γ-EC was removed when the reaction solution was exchanged at the end of the individual repeated reactions. Therefore, the decrease in both the reaction rate and yield could be caused by a decrease in NsPCS activity. Inactivation and/or release from the carrier of NsPCS may explain this decrease in activity. To improve the reaction rate and yield, further improvements in immobilization and reaction conditions are required. Like the cellulose sponge, MCC is a popular immobilization carrier for enzymes and microorganisms [23, 24]. In the case of MCC, a more stable but comparable production was achieved, but the reaction rate was significantly lower than that of the cellulose sponge. We expect that the MCC should achieve stable production in more than five repeated reactions without a decrease in enzyme activity. In addition to repeated batch reactions and production at a low reaction rates, the continuous production of γ-EC could be achieved using column-packed MCC to immobilize NsPCS.

As the feasibility of large-scale production, for example, the size and shape, ratios of the weight and volume to the reaction volume, and stirring conditions employed in this study would be reproducible when increasing the reaction volume to 1,000 mL for repeated batch reactions of NsPCS immobilized on both the cellulose sponge and MCC. In 5 times batch repeated reaction with a 1,000 mL reaction volume using the sponge as the carrier, whose cost would be 2,400 USD, 61 g γ-EC would be produced, which is equivalent to approximately 226,000 USD estimated from the current market price. Therefore, our γ-EC production process using immobilized NsPCS can be performed at much lower cost and higher productivity rates with higher volumetric efficiency than current chemical production.

## Data Availability

The datasets used and/or analyzed during this study are available from the corresponding author on reasonable request.
